# Short‐term and long‐term effects of pathogen reduction interventions on salmonellosis from whole chickens

**DOI:** 10.1002/fsn3.859

**Published:** 2018-10-18

**Authors:** Thomas P. Oscar

**Affiliations:** ^1^ U. S. Department of Agriculture Agricultural Research Service Residue Chemistry and Predictive Microbiology Research Unit Center for Food Science and Technology University of Maryland Eastern Shore Princess Anne Maryland

**Keywords:** chicken, consumer resistance, food safety, pathogen reduction interventions, risk assessment, *Salmonella*

## Abstract

The current study was undertaken to evaluate short‐term and long‐term effects of pathogen reduction interventions on food safety. This was accomplished using a model that predicts risk of salmonellosis from whole chickens produced by different scenarios. Interventions investigated were a 50% pathogen reduction before retail (PR), a 50% pathogen reduction at serving by consumer education (CE), and a 75% pathogen reduction by PR + CE. Long‐term effects were simulated by reducing consumer resistance by an amount equal to reductions in pathogen exposure caused by interventions in the short‐term. In the short‐term, risk of salmonellosis was reduced (*p *<* *0.05) from 0.42 to 0.21, 0.23, and 0.13 cases per 100,000 consumers by PR, CE, and PR + CE, respectively. However, in the long‐term, risk of salmonellosis was increased (*p *<* *0.05) from 0.42 to 1.03, 1.08, and 2.20 cases per 100,000 consumers by PR, CE, and PR + CE, respectively. These results indicated that food safety benefits of pathogen reduction interventions reversed with time because of a decrease in consumer resistance to salmonellosis.

## 1 INTRODUCTION

Non‐typhoid *Salmonella* bacteria cause gastroenteritis or salmonellosis in humans. Symptoms of this disease include diarrhea, abdominal cramps, vomiting, and fever. Chickens can be carriers of *Salmonella* that infect humans (Bryan & Doyle, 1995). Consequently, the chicken industry applies multiple pathogen reduction interventions throughout the farm‐to‐table chain. On the farm, these interventions could include heat‐treated feed, pest control, litter treatments, and feed additives (Vandeplas, Dubois, Beckers, Thonart, & Thewis, 2010). In the processing plant, some interventions that could be applied are equipment sanitation, carcass washing, and antimicrobial rinses (Russell, 2012). In the distribution channel, consumer education programs that provide proper storage, handling, and cooking instructions online and on product labels are used to promote food safety.

A potential consequence of current efforts to reduce consumer exposure to *Salmonella* on chicken is that consumer resistance to salmonellosis may decline over time resulting in a rebound of salmonellosis cases. Because this scenario has important implications for food safety but has not been investigated for *Salmonella*, the current study was undertaken to evaluate short‐term and long‐term effects of pathogen reduction interventions on risk of salmonellosis from chicken. This was accomplished using a published model (Oscar, 2004b) that predicts risk of salmonellosis from whole chickens produced by different scenarios. Long‐term effects of pathogen reduction interventions on salmonellosis from whole chickens were simulated by reducing consumer resistance by an amount equal to the reduction in consumer exposure caused by interventions. Long‐term effects of interventions on salmonellosis from chicken were confirmed by simulating data from a human refeeding trial (McCullough & Eisele, 1951). Although this human refeeding trial was conducted many years ago and human immunity to *Salmonella* may have changed, to the best of the author's knowledge, this is the only human refeeding trial ever conducted with non‐typhoid *Salmonella*.

## 2 MATERIALS AND METHODS

### 2.1 Model description

The model used to predict risk of salmonellosis from whole chickens produced by different scenarios was developed and then was validated against epidemiological data as previously described (Oscar, 2004b). In brief, the model was created in a computer spreadsheet (Excel, Microsoft Corporation, Redmond, WA) and was simulated using a spreadsheet add‐in program (@Risk, Palisade Corporation, Ithaca, NY). Input settings for the baseline scenario (Table 1) were established using existing data and models. The model consisted of five unit operations and associated (pathogen events): (a) retail (contamination); (b) consumer transport (growth);(c) cooking (death); (d) serving (cross‐contamination); and (e) consumption (dose‐response). Pathogen events were simulated as rare events by linking a discrete distribution for incidence of the event with a continuous (pert) distribution for extent of the event. This was done to simulate both non‐contaminated and contaminated chickens together. Dose‐response for individual consumption events was simulated as a discrete event with two possible outcomes: no illness or illness. Here, an illness dose was randomly assigned to each chicken simulated, and if the dose consumed was less than the illness dose, then no illness occurred; otherwise, an illness occurred. No assumptions were made about the mechanism that accounted for a particular illness dose. For example, a low illness dose (e.g., 10 cells) could be due to a high‐risk consumer or a high‐risk strain of *Salmonella* or a high‐risk meal or two or all three of these possibilities.

**Table 1:** Input settings in the model for predicting the risk of salmonellosis from whole chickens: baseline scenario

				Extent			
Node	Unit operation	Pathogen event	Incidence (%)	Minimum	Median	Maximum	Units
1	Retail	Contamination	30	0	1	2.7	log MPN/chicken
2	Transport	Growth	0.02	0.0005	0.04	0.15	log change/chicken
3	Cooking	Death	100	−96	−8.1	−0.83	log change/chicken
4	Serving	Cross‐contamination	28	0.021	0.057	0.024	Transfer coefficient
5	Consumption	Dose‐Response	100	1	3	7	log MPN

### 2.2 Scenario analysis

The first set of scenarios simulated short‐term effects of pathogen reduction interventions on risk of salmonellosis from whole chickens. Pathogen reduction before retail (PR) was simulated by reducing incidence of *Salmonella* contamination at retail from 30% in the baseline or no intervention (none) scenario (Table 1) to 15%. Pathogen reduction at serving by consumer education (CE) was simulated by reducing incidence of *Salmonella* cross‐contamination during serving from 28% in the baseline scenario (Table 1) to 14%. The combination of PR and CE (PR + CE) was simulated by reducing incidence of *Salmonella* contamination at retail from 30% in the baseline scenario to 15% and by reducing incidence of *Salmonella* cross‐contamination during serving from 28% in the baseline scenario to 14%.

Long‐term effects of pathogen reduction interventions on risk of salmonellosis from whole chickens were simulated by reducing illness dose by an amount equal to the amount of pathogen reduction obtained by the PR, CE, and PR + CE interventions. This assumption was made to provide equivalence between decreased exposure to *Salmonella* and decreased consumer resistance to *Salmonella* so as not to bias these scenarios toward one or the other direction as there are no data available to quantify the relationship between decreased exposure to *Salmonella* and decreased consumer resistance to *Salmonella*.

**Figure 1:**
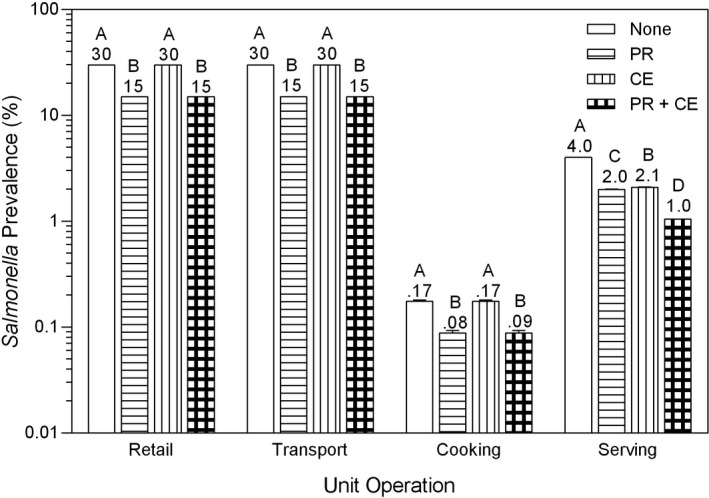
Prevalence of *Salmonella* on whole chickens (None) as affected by pathogen reduction interventions before retail (PR), at serving by consumer education (CE), and by PR + CE. Bars (mean ± *SD*) within a unit operation with different letters differ at *p *<* *0.05. The numbers above the bars are the prevalence of *Salmonella*

**Figure 2:**
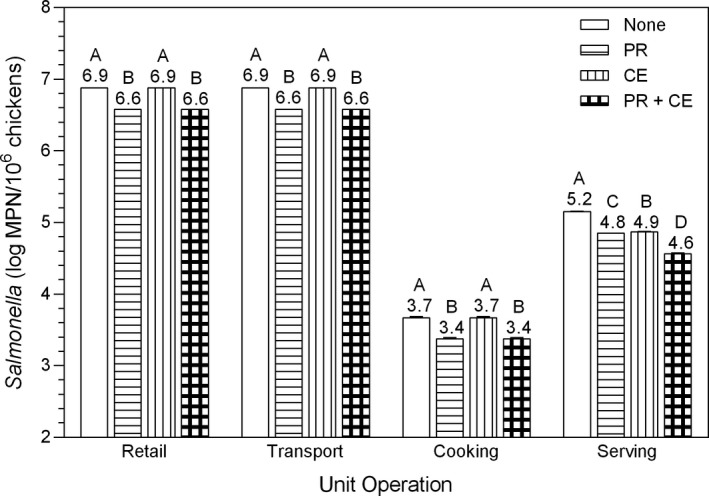
Total log most probable number (MPN) of *Salmonella* on whole chickens (None) as affected by pathogen reduction interventions before retail (PR), at serving by consumer education (CE), and by PR + CE. Bars (mean ± *SD*) within a unit operation with different letters differ at *p *<* *0.05. The numbers above the bars are the total log number of *Salmonella* per one million chickens

**Figure 3:**
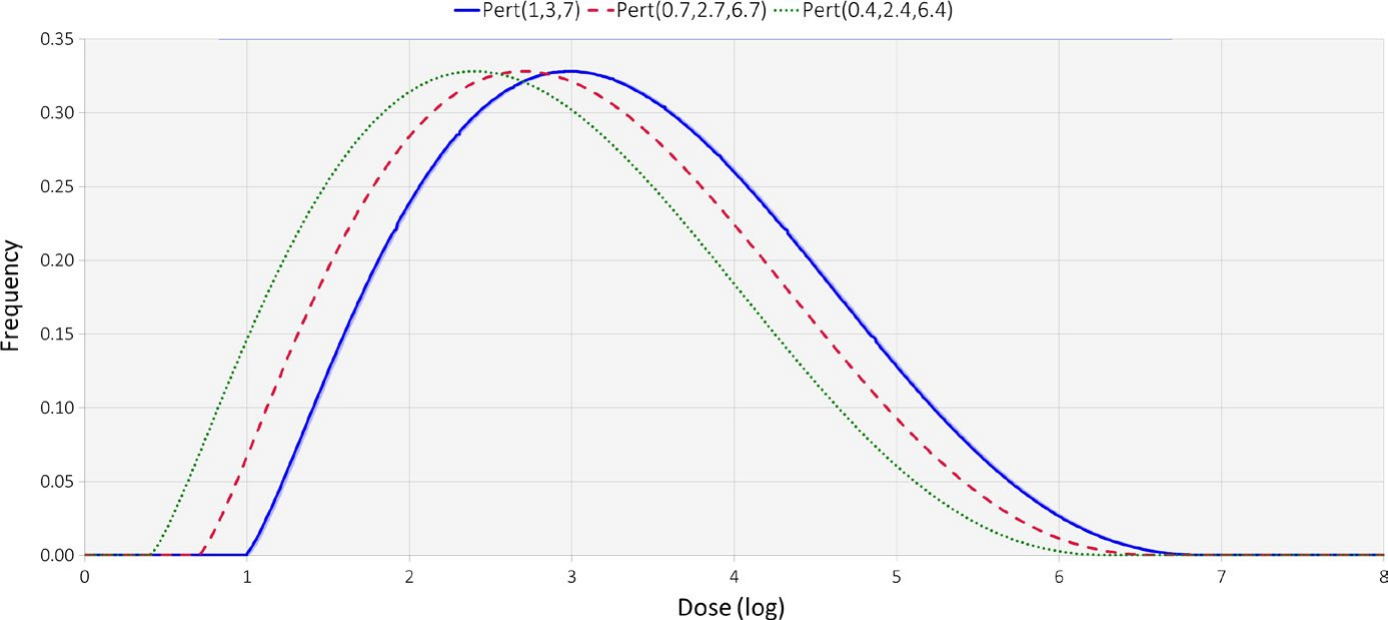
Pert distributions used to simulate changes in consumer resistance to *Salmonella*: pert (1, 3, 7) for scenario “None,” pert (0.7, 2.7, 6.7) for scenarios “PR” and “CE,” and pert (0.4, 2.4, 6.4) for scenario “PR + CE.”

The PR intervention and the CE intervention reduced consumer exposure by 50%, whereas the PR + CE intervention reduced consumer exposure by 75% (Figures 1 and 2). Consequently, to simulate the decrease in consumer resistance to *Salmonella* from decreased exposure to *Salmonella*, the pert distribution for illness dose (Table 1 and Figure 3) was reduced from 1 (minimum), 3 (median), and 7 (maximum) log in the baseline scenario to 0.7 (minimum), 2.7 (median), and 6.7 (maximum) log in the PR and CE scenarios and to 0.4 (minimum), 2.4 (median), and 6.4 (maximum) log in the PR + CE scenario.

### 2.3 Confirmation of results

To confirm long‐term effects of pathogen reduction interventions on risk of salmonellosis from whole chickens, results from a human refeeding trial (McCullough & Eisele, 1951) were simulated. In the human refeeding trial, subjects were refed a higher dose of the strain of *Salmonella* that caused them to become ill in the original feeding trial. An increase in resistance to salmonellosis after refeeding was observed in the form of a milder illness or no illness after being fed the higher dose of *Salmonella*. The difference between the original dose of *Salmonella* fed that caused salmonellosis and the refed dose of *Salmonella* that caused no illness, or a milder illness was used as a conservative measure of the change in consumer resistance to salmonellosis (Table 2).

**Table 2:** Results of a trial with human subjects fed and refed *Salmonella* to assess the increase in resistance from prior exposure

Human subject	*Salmonella* strain	Original dose (log)	Time between feedings (months)	Refed dose (log)	Response	Original dose ‐ refed dose (log)
55	Meleagridis I	7.380	9.0	7.592	No illness	0.212
59	Meleagridis I	7.699	8.5	7.893	Mild illness	0.194
60	Meleagridis I	7.699	8.5	7.893	No illness	0.194
61	Meleagridis I	7.699	8.5	7.893	Mild illness	0.194
77	Meleagridis II	7.000	6.5	7.179	Mild illness	0.179
90	Meleagridis II	7.613	4.5	7.862	Mild illness	0.249
91	Meleagridis II	7.613	4.5	7.862	No illness	0.249
92	Meleagridis II	7.613	4.5	7.862	No illness	0.249
93	Meleagridis II	7.613	4.5	7.862	No illness	0.249
112	Meleagridis III	6.885	3.0	7.090	No illness	0.205
115	Meleagridis III	7.000	1.8	7.090	No illness	0.090
116	Meleagridis III	7.000	2.0	7.176	No illness	0.176
162	Anatum I	5.934	9.0	6.114	Mild illness	0.179
163	Anatum I	5.934	9.0	6.114	Mild illness	0.179
164	Anatum I	5.934	9.0	6.114	No illness	0.179
211	Anatum II	7.827	1.8	7.976	No illness	0.149
212	Anatum II	7.827	1.8	7.976	No illness	0.149
213	Anatum II	7.827	1.8	7.976	No illness	0.149
214	Anatum II	7.827	1.8	7.976	No illness	0.149
228	Anatum III	6.669	3.0	6.799	No illness	0.130
230	Anatum III	6.669	3.5	6.908	No illness	0.239
231	Anatum III	6.669	3.0	6.799	Mild illness	0.130
233	Anatum III	6.669	3.0	6.799	No illness	0.130

In reality, the change in consumer resistance was underestimated because the dose that caused prior illness was equal to or more than the dose needed to cause illness and the refed dose was less than the current dose needed to cause illness because none of the refed doses caused illness. Nonetheless, the minimum, median, and maximum increase in illness dose, which again was underestimated for the aforementioned reasons, in the human refeeding trial were 0.09, 0.18, and 0.25 log, respectively (Table 2). These values were used to define a new pert distribution in the model whose output was subtracted from the output of the pert distribution for illness dose. This was done to simulate the heterogeneity of the loss of consumer resistance from less previous exposure to *Salmonella*. Here, it was assumed that the loss of consumer resistance to *Salmonella* from less exposure to *Salmonella* was equivalent to the gain in consumer resistance to *Salmonella* from prior exposure to *Salmonella* in the refeeding trial. This simulation was done to confirm the results from the long‐term pathogen reduction scenarios. Namely, to show, based on an approximation from actual data, what a loss of consumer resistance to *Salmonella* from less exposure to *Salmonella* would have on the rate of salmonellosis from chicken.

### 2.4 Model simulation

To evaluate short‐term effects of pathogen reduction interventions on risk of salmonellosis from whole chickens, four scenarios were simulated as follows: (a) baseline or none (Table 1); (b) PR; (c) CE; and (d) PR + CE. To evaluate long‐term effects of pathogen reduction interventions on risk of salmonellosis from whole chickens, a second set of four scenarios was simulated as follows: (a) baseline or none (Table 1); (b) PR with 50% reduced consumer resistance; (c) CE with 50% reduced consumer resistance; and (d) PR + CE with 75% reduced consumer resistance. To confirm long‐term effects of pathogen reduction interventions on risk of salmonellosis from whole chickens, two additional scenarios were simulated as follows: (a) baseline or none (Table 1); and (b) baseline or none with reduced consumer resistance based on a human refeeding trial (McCullough & Eisele, 1951), as described above. The baseline or none scenario was the control scenario where no intervention was applied.

All scenarios were simulated with @Risk settings of Latin Hypercube sampling, 10^6^ iterations, and random number generator seeds of 1, 7, 29 and 83 per the design of the original study (Oscar, 2004b). The random number generator seed is a number that initiates the selection of random numbers by the spreadsheet add‐in program (@Risk). Each random number generator seed produces a unique outcome of the model.

### 2.5 Data analysis

Results were calculated per the original study (Oscar, 2004b). In brief, results of the model simulations were filtered to remove results for non‐contaminated chickens and then the prevalence (%) of *Salmonella* after each unit operation was calculated by dividing the number of contaminated chickens by 10^6^ and multiplying by 100 to obtain %. Next, the mean number of *Salmonella* per contaminated chicken was multiplied by the number of contaminated chickens and log base 10 transformed to obtain the total log number of *Salmonella* per 10^6^ chickens after each unit operation. Finally, it was assumed that four people ate each chicken and that one of them consumed all the *Salmonella*. Thus, cases of salmonellosis per 10^6^ chickens were divided by 40 (4 consumers × [10^6^/10^5^]) to obtain the cases of salmonellosis per 100,000 consumers.

### 2.6 Statistical analysis

Two‐way analysis of variance with seed as a blocking factor was used to evaluate effects of pathogen reduction interventions, unit operations, and their interaction on prevalence and total log number of *Salmonella* on whole chickens. When analysis of variance was significant (*p *<* *0.05), means were compared among pathogen reduction interventions within unit operations using Tukey's multiple comparison test at *p *<* *0.05.

Two‐way analysis of variance with seed as a blocking factor was used to evaluate effects of pathogen reduction interventions, duration of effects (short‐term or long‐term), and their interaction on rate of salmonellosis from whole chickens. When analysis of variance was significant (*p *<* *0.05), means among pathogen reduction interventions were compared within duration of effects using Tukey's multiple comparison test at *p *<* *0.05.

A paired student's *t* test (*p *<* *0.05) with seed as the pairing factor was used to compare rate of salmonellosis from whole chickens in the baseline scenario to rate of salmonellosis from whole chickens in the baseline scenario with reduced consumer resistance that was based on results from a human refeeding trial (McCullough & Eisele, 1951). All statistical analyses were performed using GraphPad Prism version 6.00 for Windows (GraphPad Software Inc., La Jolla, CA).

## 3 RESULTS

### 3.1 *Salmonella* prevalence

In the short‐term, the prevalence of *Salmonella* on whole chickens was affected (*p *<* *0.05) by a pathogen reduction intervention by unit operation interaction (Figure 1). The PR intervention reduced (*p *<* *0.05) *Salmonella* prevalence by 50% throughout the retail‐to‐serving chain, whereas the CE intervention only reduced (*p *<* *0.05) *Salmonella* prevalence by 50% at serving. These results were expected because the PR intervention was applied before retail and the CE intervention was applied after cooking. When the PR and CE interventions were applied together, *Salmonella* prevalence was reduced (*p *<* *0.05) by 75% at serving from 4% for no intervention (None) to 1% for the PR + CE intervention. These results were the same for long‐term effects of pathogen reduction interventions on prevalence of *Salmonella* because the input settings from retail‐to‐serving were the same for both sets of scenarios.

### 3.2 *Salmonella* number

In the short‐term, total log number of *Salmonella* per 10^6^ chickens was affected (*p *<* *0.05) by a pathogen reduction intervention by unit operation interaction (Figure 2). Here, the PR intervention reduced (*p *<* *0.05) the total number of *Salmonella* by 0.3 log (50%) at retail, after consumer transport, and after cooking but reduced the total number of *Salmonella* by 0.4 log (60%) at serving. On the other hand, the CE intervention, which was applied at serving, as expected, did not reduce the total log number of *Salmonella* before serving but reduced (*p *<* *0.05) the total number of *Salmonella* at serving by 0.3 log (50%). When the PR and CE interventions were applied together, the total number of *Salmonella* consumed was reduced (*p *<* *0.05) by 0.6 log (75%) at serving. These results were the same for long‐term effects of pathogen reduction interventions of the total log number of *Salmonella* because the input settings from retail‐to‐serving were the same for both sets of scenarios.

### 3.3 Risk of salmonellosis

**Figure 4:**
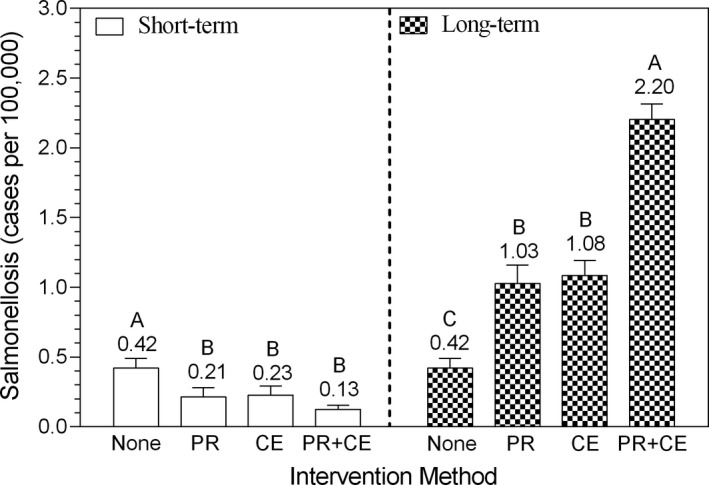
Rate of salmonellosis from whole chickens (None) as affected by pathogen reduction interventions before retail (PR), at serving by consumer education (CE), and by PR + CE. Bars (mean ± *SD*) within short‐term or long‐term effect categories with different letters differ at *p *<* *0.05

Having achieved a reduction in consumer exposure to *Salmonella* in the short‐term and long‐term with the PR, CE, and PR + CE interventions, the next step was to evaluate the short‐term and long‐term effects of these pathogen reduction interventions on the risk of salmonellosis (Figure 4). In the short‐term, the rate of salmonellosis (cases/100,000 consumers) was reduced (*p *<* *0.05) from 0.42 ± 0.07 (mean ± *SD*) to 0.21 ± 0.07, 0.23 ± 0.06, and 0.13 ± 0.03 for the PR, CE, and PR + CE interventions, respectively. However, in the long‐term, when resistance of consumers to salmonellosis was reduced in proportion to the reduction in consumer exposure to *Salmonella* in the short‐term, the rate of salmonellosis (cases/100,000 consumers) increased (*p *<* *0.05) from 0.42 ± 0.07 to 1.03 ± 0.13, 1.08 ± 0.11, and 2.20 ± 0.11 for the PR, CE, and PR + CE interventions, respectively.

### 3.4 Refeeding trial simulation

To confirm results for long‐term effects of pathogen reduction interventions on the risk of salmonellosis from whole chickens, the baseline scenario was simulated with illness dose adjusted for changes in consumer resistance that were based on results of a human refeeding trial (McCullough & Eisele, 1951), as described above. Here, the rate of salmonellosis (cases/100,000 consumers) increased (*p *<* *0.05) from 0.42 ± 0.07 for the baseline scenario to 1.21 ± 0.29 for the human refeeding trial scenario indicating that a shift of the dose‐response downward or to the left (i.e., less consumer resistance) will result in an increase of cases of salmonellosis, thus confirming the results of the scenarios for long‐term effects of pathogen reduction interventions on the risk of salmonellosis.

## 4 DISCUSSION

A published retail‐to‐table model (Oscar, 2004b) was used in the present study to evaluate the short‐term and long‐term effects of pathogen reduction interventions applied throughout the farm‐to‐table chain on the risk of salmonellosis from whole chickens. The model was actually a farm‐to‐table model because the distribution of *Salmonella* contamination on whole chickens at retail was a summation of all unit operations and associated pathogen events that occur red before retail. Thus, the model can be used to evaluate effects of pathogen reduction interventions that are applied throughout the chicken production chain: in the hatchery, at the feed mill, on the grow‐out farm, in the processing plant, and/or in the distribution channel before retail. This can be accomplished by acquiring data for *Salmonella* contamination at retail for whole chickens produced by the baseline or control scenario and by acquiring data for *Salmonella* contamination at retail for whole chickens produced by the pathogen reduction intervention or test scenario.

In the current study, effects of a pathogen reduction intervention being applied before retail were simulated by reducing incidence of *Salmonella* contamination at retail from 30% for the baseline or control scenario to 15% for the pathogen reduction intervention or test scenario. The specific intervention applied was not identified but it could have been the following: (a) application of a competitive exclusion product in the hatchery (Mead, 1990); (b) heat treatment of feed at the feed mill (Maciorowski, Jones, Pillai, & Ricke, 2004); (c) a litter treatment on the grow‐out farm (Payne, Kroger, & Watkins, 2002); (d) an antimicrobial rinse in the processing plant (Kim et al., 1994), or (e) high pressure treatment of the packaged product just before distribution from the processing plant (Demazeau & Rivalain, 2011).

Although it is possible to develop a model that includes unit operations and associated pathogen events before retail, acquiring data for pathogen contamination at multiple points in the food production chain is time consuming and expensive. A more cost‐effective approach is that used in the present study, namely, to obtain data for pathogen contamination at one point in the food production chain (e.g., retail) and then use modeling to forecast how that contamination could change from that point forward.

When a consumer is exposed to a dose of pathogen in their food, their response ranges from no‐response‐to‐infection to mild‐illness‐to‐illness to sever‐illness‐to‐death. To model the consumer response of interest (e.g., illness), criteria are used to place the response into one of the aforementioned categories (Oscar, 2004a). It should be noted that exposure to a pathogen like *Salmonella* does not result in a probability of illness; rather, the consumer either becomes ill or they do not.

In the current study, the response modeled was illness, which occurred when the infection was severe enough to result in a doctor visit and diagnosis of salmonellosis. This response was modeled for two reasons: first, to compare model predictions to epidemiological data for diagnosed cases of salmonellosis. For the baseline scenario, the predicted rate of illness was 0.42 cases of salmonellosis per 100,000 consumers of chicken as compared to 0.66–0.88 cases of salmonellosis per 100,000 consumers of chicken as predicted by epidemiological data (Bryan & Doyle, 1995; Oscar, 2004b); second, once the rate of illness is predicted by the model, epidemiological data can be used to calculate severity of illness. For *Salmonella*, the percentage of ill patients that are hospitalized is 27.2%, whereas the percentage of ill persons that die is 0.5% (Scallan et al., 2011). Thus, the rate of hospitalization for the baseline scenario in this study was 0.11 cases per 100,000 consumers of chicken, whereas the rate of mortality for the baseline scenario in the present study was 0.0021 cases per 100,000 consumers of chicken.

Current efforts to improve food safety are focused on reducing consumer exposure to pathogens of food origin without consideration of the long‐term impact of pathogen reduction interventions on consumer resistance to salmonellosis. In the present study, it was demonstrated that a reduction in consumer resistance that was equal to the reduction in consumer exposure from interventions resulted in more cases of salmonellosis from chicken in the long‐term than if no pathogen reduction interventions had been applied. The importance of consumer resistance in the long‐term control of salmonellosis was confirmed by simulating actual data on consumer resistance to salmonellosis that was obtained from a human refeeding trial (McCullough & Eisele, 1951). These results agree with previous studies (Havelaar & Swart, 2014; Swart, Tomasi, Kretzschmar, Havelaar, & Diekmann, 2012) conducted with another pathogen, *Campylobacter*, commonly found on chicken where increases in consumer immunity were shown to reduce risk of campylobacteriosis.

Together these results indicate that current efforts to improve food safety by reducing consumer exposure to *Salmonella* from chicken might actually increase the rate of salmonellosis in the long‐term due to a reduction in consumer resistance to this pathogen. Thus, consideration should be given to expanding food safety efforts to include interventions, such as vaccination, proper nutrition, and stress reduction that maintain or enhance consumer resistance to pathogens. Computer models, such as the current one, can help define the proper balance needed between consumer exposure and consumer resistance to foodborne pathogens for maximizing public health.

In the Australian meat and poultry industry, the prevalence of *Salmonella* on red meat and poultry declined by over 50% from 1994 to 2004 but the rate of salmonellosis in the human population stayed the same (Sumner, Raven, & Givney, 2004). Lack of control over post‐processing risk factors, such as food handling practices, and increased salmonellosis from non‐food sources, such as water, pets, and farm animals, were offered as possible explanations for this observation. A reduction of consumer resistance to salmonellosis because of reduced exposure to *Salmonella* as simulated in the present study is also consistent with a steady rate of salmonellosis while levels of this pathogen on red meat and poultry decline. Thus, conclusions of this study are consistent with observations made in the meat and poultry industry of Australia.

## 5 CONCLUSIONS

The importance of consumer resistance in food safety has been overlooked with the unintended consequence that the current approach to food safety (i.e., reduce consumer exposure to pathogens) may actually result in more rather than less foodborne illness in the long‐term. Thus, consumer resistance to pathogens deserves more consideration in the future approach to food safety.

## CONFLICT OF INTEREST

The author has no conflict of interests to report.

## ETHICAL STATEMENT

This research did not require use of animal or human subjects.
